# PHB Processability and Property Improvement with Linear-Chain Polyester Oligomers Used as Plasticizers

**DOI:** 10.3390/polym14194197

**Published:** 2022-10-06

**Authors:** Rogerio Ramos de Sousa Junior, Carlos Alberto Soares dos Santos, Nathalie Minako Ito, Airton Nizetti Suqueira, Maximilian Lackner, Demetrio Jackson dos Santos

**Affiliations:** 1 Center for Engineering, Modeling and Applied Social Sciences, Federal University of ABC, Santo André 09210-580, Brazil; 2 Department of Industrial Engineering, University of Applied Sciences Technikum Wien, Hoechstaedtplatz 6, 1200 Vienna, Austria or; 3 Circe Biotechnologie GmbH, Kerpengasse 125, 1210 Vienna, Austria

**Keywords:** biopolymers, plasticizer, thermal properties, mechanical properties, PHB (polyhydroxybutyrate), PHA (polyhydroxyalkanoates)

## Abstract

In 2021, global petroleum-based plastic production reached over 400 million metric tons (Mt), and the accumulation of these non-biodegradable plastics in the environment is a worldwide concern. Polyhydroxybutyrate (PHB) offers many advantages over traditional petroleum-based plastics, being biobased, completely biodegradable, and non-toxic. However, its production and use are still challenging due to its low deformation capacity and narrow processing window. In this work, two linear-chain polyester oligomers were used as plasticizers to improve the processability and properties of PHB. Thermal analyses, XRD, and polarized optical microscopy were performed to evaluate the plasticizing effect on the PHB and the reflection on the mechanical behavior. Both oligomers acted as PHB plasticizers, with a reduction in T_g_ and T_m_ as a function of the plasticizer concentration, which can make it easier to handle the material in thermal processing and reduce the probability of thermal degradation. Plasticizer 2 proved to be the most promising between the two with an optimized condition of 20%, in which there was a decrease in elastic modulus of up to 72% and an increase in the maximum elongation of 467%.

## 1. Introduction

In 2021, global plastic production exceeded 400 million metric tons (Mt), and around 85 percent of the total was petroleum-derived [1]. While petroleum-based plastics are essential to virtually all industrial segments, the accumulation of these non-biodegradable plastics in marine, freshwater, and terrestrial ecosystems is a global concern [2,3,4]. In this context, researchers have focused their attention on developing and producing biodegradable plastics.

Polyhydroxyalkanoates (PHAs) are biodegradable polymers that are partially crystalline, with a comparatively high melting temperature (T_m_ ≈ 175 °C) and a high degree of crystallinity classically produced from different types of microorganisms, such as Alcaligenes, Azobacter, Bacillus, and Pseudomonas. These ferment organic matter and accumulate PHA in the presence of carbon and other nutrient-deficient conditions (typically through N or P limitation) [5,6,7,8]. Cyanobacteria have been reported to synthesize PHB from CO_2_ as a sole carbon source [9,10]. Methanotrophic bacteria can yield PHB from CH_4_ as a sole carbon and energy source [11,12]. Thereby, the latter two classes of microorganisms do not require feed or food sources such as glucose for PHB production and can offer a sustainable route for scale-up.

Polyhydroxybutyrate (PHB) is the most common form of PHA. It is a naturally occurring compound. PHB has thermoplastic properties and offers many advantages over traditional petroleum-based plastics, being completely biodegradable and non-toxic, with good gas barrier and mechanical properties similar to polypropylene, and a similarity in thermal properties and tensile strength to polyesters [7,13,14,15,16]. Hence, PHB is a promising candidate to replace non-biodegradable plastics in many applications such as food services, biomedicine, and product packaging [13,14,15]. Its copolymers such as PHBV and blends with other (bio)polymers can extend the application range.

Nevertheless, the manufacture of large-scale PHB products is still a challenge, as it is a material of high stiffness and low deformation capacity, and is very sensitive to thermal processing conditions, with a narrow processing window of processability in the molten state. A high processing temperature (close to or above T_m_) is required, which can cause thermal degradation, resulting in a decrease in molecular weight and a further reduction in the melt strength of the crystalline domains [17,18].

In principle, this problem can be overcome by adding low-molecular-weight compounds to the PHB, acting as eco-environmentally friendly external plasticizers. Traditionally, the addition of plasticizers makes it possible to reduce the glass transition temperature (T_g_) and the average melting temperature (T_m_), which facilitates thermal processing and reduces the probability of thermal degradation. In addition, such compounds provide flexibility to the polymer structure and there is an increase in structural spacing, causing greater toughness and flexibility to the material [7,17,19,20].

In recent years, different plasticizers have been used with these biopolymers. These can be classified as low molecular weight, such as citrates, glycerol, plant oils, and esters, or oligomeric plasticizers (M_n_ 1000–6000 g mol^−1^ [21]), such as polyethylene glycol, propylene glycol, polyisobutylene, and aliphatic polyesters [22,23,24,25,26,27]. The most effective plasticizers are those with low molecular weight and groups available for interaction with the polymer matrix [25]. They present good miscibility in the PHB matrix, with a reduction in T_g_, an increase in impact strength, and maximum elongation at break.

However, there is also the occurrence of mass loss at temperatures in the processing range [19,24] and the migration of these additives to the surface of the PHB, which results in a deterioration of the material’s physical properties. As an alternative, oligomeric plasticizers have been used. Although they have lower mixing entropy when compared to those with lower molecular weight, they are more thermally stable and, due to low chain mobility, less prone to migration [28]. Aliphatic polyester oligomers have great potential as PHB plasticizers [25]. Obtained from the polycondensation reaction of dicarboxylic acids and diols, they are biodegradable materials [29] and have been applied in packaging, agriculture, and bio-medicine industries [30].

Frone et al. [28] evaluated poly(3-hydroxyoctanoate) (PHO) and tributyl 2-acetyl citrate (TAC) as plasticizers in PHB in concentrations up to 20 wt%. Both resulted in a decrease in elastic modulus and an increase in elongation at break, in addition to a reduction in T_g_, with a more pronounced effect for the TAC of a lower molecular weight. They also observed the migration of TAC to the surface, which did not occur with the addition of higher-molecular-weight PHO.

In this work, two linear-chain polyester oligomers—plasticizer 1 and plasticizer 2 (P1 and P2, respectively)—were used to evaluate the plasticizing effect on the PHB matrix at concentrations up to 30 wt%. The plasticizers are distinguished by the C-O/C=O ratio: approx. 0.923 and 2.226 for P1 and P2, respectively. Thermogravimetric analyses were performed to verify the thermal stability of plasticized PHB; moreover, the main thermal transitions and the crystallinity index were confirmed by DSC. These results were used in conjunction with XRD and morphological analyses of the systems to determine the interaction of plasticizers in the polymer chain, making it possible to evaluate the effects of miscibility and variation in the nucleation/growth rate of spherulites as a function of each plasticizer. Finally, plasticized PHB formulations were subjected to mechanical tensile tests to determine the effect of plasticizers on elastic modulus, tensile strength, and elongation at break. In this way, it was possible to determine that the plasticizer P2 has a better plasticizing effect for PHB, due to greater spacing in the polymer matrix, and that both plasticizers have a critical concentration of 20 wt%; higher concentrations cause phase separation and consequently mechanical deterioration. Thus, at the limit of mixture miscibility, the addition of oligomer plasticizers provides effective plasticization with greater thermal stability than low-molecular-weight plasticizers.

## 2. Materials and Methods

### 2.1. Materials

The PHB in this study was obtained from the cyanobacterial strain *Synechocystis* sp. PCC 6714 feeding on CO_2_ as a sole carbon source.

An axenic culture of wild-type strain *Synechocystis* sp. PCC 6714 was purchased from the Pasteur Culture Collection of Cyanobacteria (Pasteur Institute, Paris, France). The cells were grown in a modified BG-11 medium at pH 8.2. In order to induce nitrogen deficiency, cells were cultured in BG-11 medium without nitrate and ammonia. (NH_4_)_5_[Fe(C_6_H_4_O_7_)_2_] and Co(NO_3_)_2_·6H_2_O were substituted with equimolar concentrations of FeC_6_H_5_O_7_ and CoCl_2_·6H_2_O with regard to iron and cobalt content. For phosphorus limitation, KH_2_PO_4_ was replaced with an equimolar concentration of KCl for potassium content [10].

The highest average volumetric PHB production rate was obtained during two-step cultivation with a value of 14 mg L^−1^ d^−1^, and the highest specific PHB production rate was determined during a one-step process with a value of 5.4 mg g^−1^ d^−1^. The strain could produce up to 16% (DCW) PHB under nitrogen and phosphorous limitation [9].

The pilot-scale cultivations were performed in a 40 L glass reactor of tubular with a vertical design (airlift). The circulation was performed using sterile filtered air [31].

For PHB extraction, the biomass was lyophilized and suspended in chloroform at 30 mL g^−1^ of biomass. The suspension was put on a heating block and allowed to boil for one hour under continuous shaking at 300 rpm. The hot suspension was filtered through filter paper. The PHB was extracted using 10 times volume of ice-cold methanol. The polymer was separated using centrifugation at 30,000 rpm, and then was air-dried. The PHB was finally washed using cold acetone [31].

The GPC analysis showed that the molecular weight was (Mw = 1,051,900 g mol^−1^) and the number average of the molecular weight of the PHB was (Mn = 316,060 g mol^−1^). The polydispersity index (PDI—M_w_/M_n_) of the cyanobacterial PHB was determined to be 3.328 [31].

Oligomers were used as received. Plasticizer 1 (P1) was derived from the reaction of adipic acid (C_6_H_10_O_4_), propylene glycol (C_3_H_8_O_2_) and lactic acid (C_3_H_6_O_3_), resulting in a material with an average molecular weight (M_n_) of 2654 g mol^−1^ and a PDI of 1.9. Plasticizer 2 (P2) was derived from the reaction of adipic acid (C_6_H_10_O_4_), ethylene glycol (C_2_H_6_O_2_), 1,4-butanediol (C_4_H_10_O_2_), and 2-ethyl-1-hexanol (C_8_H_18_O), resulting in a material with M_n_ of 2382 g mol^−1^ and a PDI of 1.97. Chloroform (99% purity, supplied by LabSynth, Diadema, Brazil) was used without any further treatment.

### 2.2. X-ray Photoelectron Spectroscopy

The plasticizer surfaces were analyzed by X-ray photoelectron spectroscopy (XPS) using a K-alpha+ spectrometer (ThermoFisher Scientific, Waltham, MA, USA) with Al-kα radiation (1486.6 eV) and a pass energy of 200 eV for the survey and 50 eV for the high-resolution spectra. A flood gun was used for static charge compensation. The X-ray beam size was 400 µm. The operation was carried out at a base pressure of 10^−7^ Pa. The background was subtracted according to the Shirley model, and the peak fit was performed with a product of Gaussian and Lorentzian shapes. Atomic concentration was based on Scofield sensitivity factors [32].

### 2.3. Preparation of Plasticized PHB

The plasticized PHB samples were obtained via solution using chloroform as solvent, mixing PHB with different weight percentages (wt%) of either P1 or P2 (10, 20 and 30%). Each solution was prepared at a concentration of 0.2 g mL^−1^ and heated to 40 °C under continuous agitation for 2 h, including a solution of neat PHB. The solutions were then poured into Petri dishes and kept in a fume hood until the complete evaporation of the solvent had taken effect. The plasticized PHB and neat PHB samples were obtained by pressing the remaining material in a hydraulic press with a pressure of 0.5 MPa, at a temperature of 180 °C, for 5 min. The resulting samples were identified as PHB/xxP1 or PHB/xxP2, where xx is the weight fraction of the incorporated plasticizer.

### 2.4. Thermal Characterization

Thermogravimetric analysis (TGA) was carried out using a TGA Q500 (TA instruments, Waltham, MA, USA), from room temperature to 600 °C, at a heating rate of 10 °C min^−1^, in an inert nitrogen atmosphere. Differential scanning calorimetry (DSC) measurements were carried out on a DSC Q200 equipment (TA Instruments, Waltham, MA, USA). A first heating scan from room temperature to 200 °C (isothermal for 3 min) was used to erase the thermal history of the polymer, followed by cooling to −80 °C and reheating to 210 °C. All heating and cooling cycles were performed at a rate of 20 °C min^−1^ in a nitrogen atmosphere.

The crystallinity index (*X_DSC_*) was estimated as shown in Equation (1) [33]:

(1) $$X_{DSC}\left(\%\right)=\frac{\Delta H_{m}}{\Delta H_{m}^{0}\times w_{i}}\times 100$$

where Δ*H_m_* is the PHB melting enthalpy in the sample, $\Delta H_{m}^{0}$ is the melting enthalpy for 100% crystalline PHB ($\Delta H_{m}^{0}$ = 146 J g^−1^), and *w_i_* is the weight fraction of PHB in the plasticized sample (*w_i_* = 0.9, 0.8 or 0.7).

### 2.5. Crystalline Morphology

Polarized optical microscopy (POM) was used to evaluate the effect of the plasticizer addition on the spherulitic morphology of PHB. In a heating module T95 HS (Linkam, Salfords, UK) coupled to an Axio Scope A1 optical microscope (Carl Zeiss, Oberkochen, Germany), samples in the order of 4 mg were heated to a temperature of 210 °C, kept in isotherm conditions for 3 min, and cooled at a rate of 15 °C min^−1^ to a temperature of 60 °C, where they were kept in an isotherm for 1 h.

### 2.6. X-ray Diffraction (XRD) Analysis

The crystalline structure of PHB was studied with Stadi P equipment (Stoe, Darmstadt, Germany), with Cu-K-α (λ = 0.1542 nm) in 2θ range 5°–30° with a scan rate of 1° min^−1^. Fityk software (version 1.3.1) was used to analyze the data. For comparison purposes, the crystallinity index was also calculated using the XRD analysis (*X_XRD_*) from Equation (2):

(2) $$X_{XRD}\left(\%\right)=\frac{A_{c}}{A_{c}\times A_{a}}\times 100$$

where *A_c_* is the sum of the areas under the crystalline peaks extracted from the XRD diffractogram, and *A_a_* is the area of the amorphous halo.

### 2.7. Mechanical Properties

Tensile strength (σ), elastic modulus (E), and the elongation at break (ε) were determined using an universal testing machine model 3369 (Instron, Norwood, MA, USA). The samples were stored in a desiccator for 15 days before performing the analyses and cut into rectangles of 6.0 × 0.8 mm. Tests were carried out in ambient conditions with a crosshead speed of 2 mm min^−1^, using a gauge length of 10 mm.

## 3. Results and Discussion

### 3.1. Structure of the Polyesters

**Figure 1:**
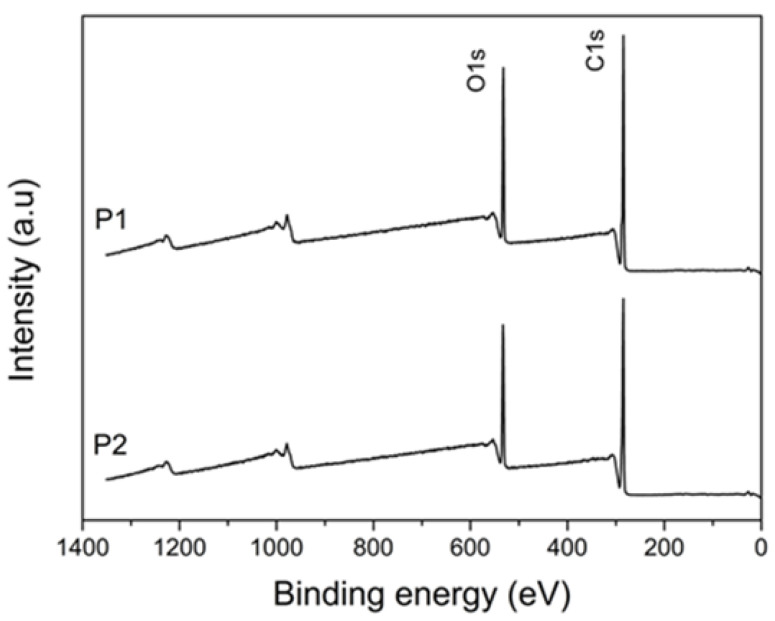
XPS spectra of plasticizers P1 and P2.

**Figure 2:**
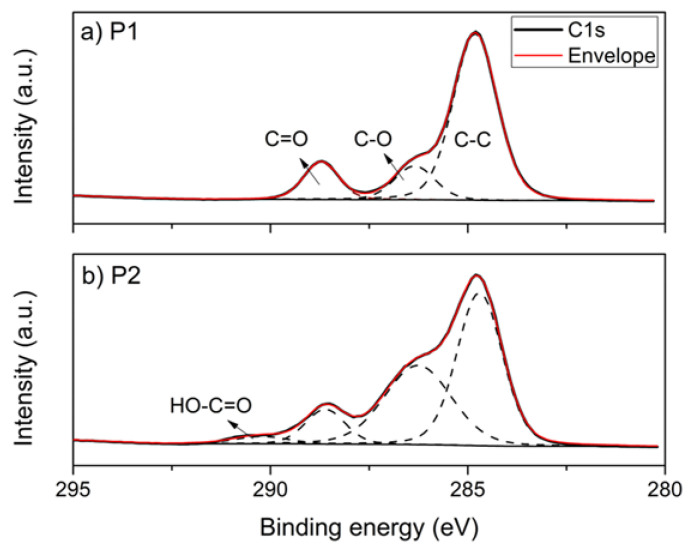
Resolved XPS spectra of C(1s) for (**a**) P1 and (**b**) P2.

**Figure 3:**
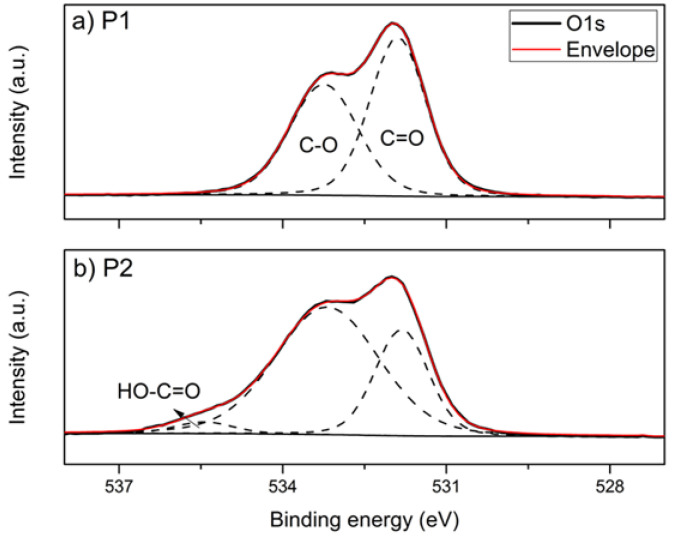
Resolved XPS spectra of O(1s) for (**a**) P1 and (**b**) P2.

XPS analyzes were performed on the plasticizers to obtain information on the surface composition of the polyesters [34]. Figure 1 shows the XPS spectra of the plasticizers, P1 and P2, where the photoemissions of C(1s) and O(1s) are observed. The high-resolution resolved spectra of C(1s) and O(1s) are shown in Figure 2 and Figure 3, respectively. The atomic concentrations of carbon and oxygen were determined from binding energy and based on sensitivity factors. Table 1 presents the data obtained in the spectra photoemission, binding energy, area, and the atomic O/C ratio.

The high-resolution resolved spectrum of C1s from P1 (Figure 2a) shows three photoemission peaks—284.78 eV, 286.35 eV, and 288.71 eV—which correspond to the C-C, C-O, and C=O bonds, respectively. Still, for sample P1, the resolved spectrum of O1s (Figure 3a) presents two peaks, 531.91 eV and 533.26 eV, which refer to C=O and C-O, respectively. The adjusted binding energy peaks corresponded to polyester and were previously observed in the literature [35]. The resolved spectra, C1s and O1s, of sample P2 are shown in Figure 2 and Figure 3, respectively. In C1s, in addition to the peaks corresponding to C-C, C-O, and C=O, a fourth photoemission peak was observed at 290.22 eV. This fourth peak possibly corresponds to an end-of-chain bond HO-C=O, or even HO-C(O)-O, originating from the 2-ethyl-1-hexanol used as a terminating agent in obtaining P2, which due to its greater electronegativity has a greater distance eV to the C-C/C-H bond [36]. Likewise, the resolved spectrum of O1s from P2 has three peaks, the two corresponding to C=O and C-O and an additional peak at 535.21 eV.

The atomic O/C ratio of plasticizers has a slight difference—0.337 and 0.306—for P1 and P2, respectively. The main difference between them occurs in the proportion of each kind of binding energy. P1 has a ratio of approx. 52% C=O and 48% C-O, i.e., each bond involving O corresponds to an ester group. The ester group is formed in the stoichiometric reaction between dicarboxylic acid (adipic acid), diol (propylene glycol), and the carboxylic acid and hydroxyl groups present in lactic acid.

On the other hand, P2 has a higher proportion of C-O compared to C=O: approx. 69% and 31%, respectively. In this case, proportionally, there is an additional bond of the C-O type for each ester group. This occurs due to the reaction of adipic acid (dicarboxylic acid) with two diols, ethylene glycol, and 1,4-butanediol.

### 3.2. Thermal Properties

**Figure 4:**
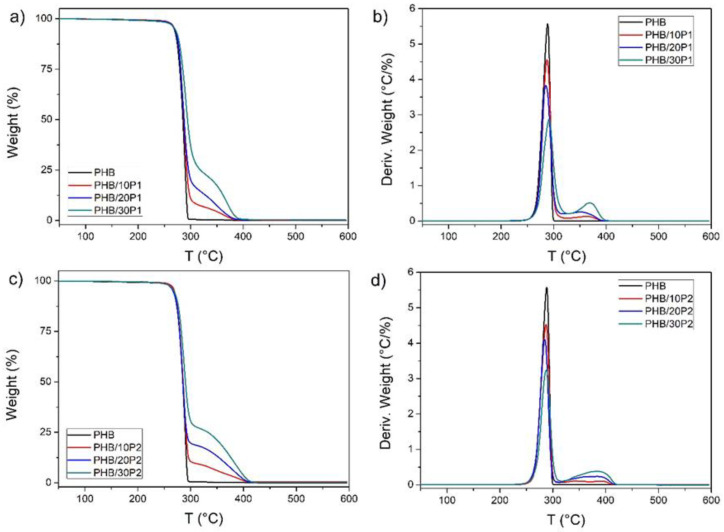
TGA (**a**,**c**) and DTG (**b**,**d**) curves of PHB, PHB/P1, and PHB/P2.

TGA and DSC analyses were carried out to evaluate the dependence of the thermal properties of PHB blends on the plasticizer used. Figure 4 shows the weight loss curves (Figure 4a,c) and their corresponding derivative curves (DTG Figure 4b,d) of neat PHB and plasticized PHB/P1 and PHB/P2. At the same time, the main thermal degradation parameters are presented in Table 2, in which it is possible to evaluate the effect of plasticizers on the thermal stability of PHB.

Neat PHB has a single weight loss event, with a temperature of the maximum degradation rate (T_d1_) at 288 °C, a value commonly observed in the literature [19,24] and which can be attributed to the random chain scission of PHB by intramolecular cis-elimination [18]. Using either P1 or P2 plasticizers did not significantly change either temperature corresponding to 5% weight loss (T_5%_ ≈ 270 °C) or the first DTG peak (T_d1_ ≈ 290 °C) in relation to PHB decomposition, respectively. The second weight loss event for plasticized PHB was around 360 °C for P1 and 390 °C for P2. The peak’s intensity is proportional to each component’s weight fraction and, therefore, can be associated with plasticizer degradation. Thus, the TGA results showed that the addition of plasticizers does not cause the degradation of PHB.

**Figure 5:**
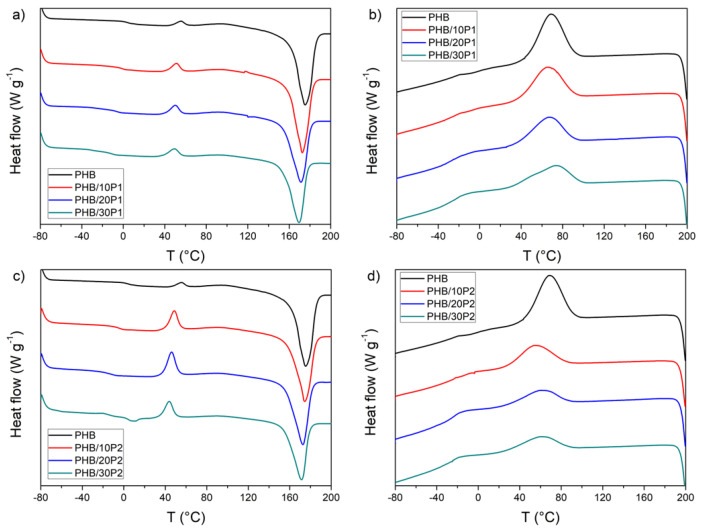
DSC thermograms corresponding to reheating (**a**,**c**) and cooling runs (**b**,**d**) of PHB, PHB/P1, and PHB/P2.

Figure 5 shows DSC curves for the second reheating cycle and the cooling cycle for the PHB plasticized with P1 (Figure 5a,b) and P2 (Figure 5c,d), respectively. The values of the main thermal parameters and X_DSC_ (calculated from Equation (1)) are summarized in Table 3.

The melting temperature peak of neat PHB was 175.24 °C. The addition of plasticizers resulted in a slight decrease in the T_m_ of the PHB blends, proportional to the concentration of each plasticizer added. The most significant reduction occurred for the addition of 30% of P1, resulting in T_m_ = 169 °C; when 30% of P2 was added, T_m_ was 171 °C. As a result of the T_m_ decrease, the processing temperature window is increased, making the PHB blends easier to process. The enthalpy of melting (ΔH_m_) of PHB was also reduced with the addition of the plasticizers.

A similar behavior was observed using the low-molecular-weight plasticizer tributyl 2-acetyl citrate (TAC) in PHB. The TAC addition induced a systematic reduction in the melting temperature values of the PHB blend, with 5–13 °C, along with its increasing content in PHB. On the other hand, in the same work, the addition of poly(3-hydroxyoctanoate) (PHO), a biosynthesized homopolymer as a plasticizer, did not induce significant changes in T_m_ due to the low influence of PHO on the mobility of PHB chains [28].

Thus, the thermal results observed in this work indicate that the plasticizers used favor the segmental movement of PHB due to the plasticizer–PHB compatibility.

As shown in Table 3, there was an increase in the degree of crystallinity in the plasticized PHB. This increase was more significant in samples with P1, showing a X_DSC_ up to 8% higher than neat PHB, while samples with P2 showed X_DSC_ up to 5% higher. The addition of plasticizers can cause either a decrease in crystallinity due to the dilution effect or its increase due to the incorporation of an additive into the amorphous phase of a semicrystalline polymer, decreasing the melting viscosity, which results in higher chain diffusion and a faster crystallization rate [19,37]. The crystallinity index values calculated for both plasticizers increased, indicating promising candidates to improve PHB processing, with a possible reduction in melt viscosity accelerating the crystallization rate.

PHB/P1 cooling curves (Figure 5b) exhibited a non-linear relationship with the plasticizer concentration. At lower concentrations, the addition of P1 causes a reduction in T_c_ compared to that of the neat PHB. However, as the P1 content increases, so does T_c_, surpassing the PHB T_c_ by 6 °C (PHB T_c_ = 68 °C, PHB/30P1 T_c_ = 72 °C). These results can be related to the interaction between PHB and P1. Both components demonstrate a complex influence on crystallization as this mixture has been reported as miscible in the molten state and partially miscible after crystallization [37]. On the other hand, adding P2 (Figure 5d) decreased T_c_ by up to 15 °C for PHB/10P2 followed by a slight increase for higher P2 concentrations, but this value was still approximately 9 °C lower than PHB T_c_. The reduction in T_c_ from the addition of P2 indicates a pronounced plasticizing effect [24], in which the interaction with the polyester oligomer possibly hinders the crystallization of PHB.

In addition, a second exothermic peak is observed in the reheating curve (Figure 5a,c). In this case, these peaks are related to cold crystallization effects and occur at temperatures (T_cc_) above the T_g_ of PHB, which allows sufficient chain mobility for crystallization to occur [19]. In both plasticizers, there was a decrease in the T_g_ of PHB, which favored the occurrence of the cold crystallization effect in the plasticized PHBs.

As both plasticizers resulted in a reduction in T_g_ and T_m_, both are seen to be good choices to improve PHB processability, with special attention given to P2, which demonstrated greater interaction in the polymer matrix.

To estimate the T_g_ of mixtures of polymers from data of the pure components and the miscibility of the mixtures, several approaches have been developed. They are commonly based on the additivity of basic thermophysical properties, and one of the most widely used equations to predict the T_g_ of amorphous mixtures and random copolymers is said to be the Fox equation. The Fox equation was used to calculate the theoretical T_g_ for each blend according to Equation (3):

(3) $$\frac{1}{T_{g}}=\frac{w_{PHB}}{T_{g\;PHB\;}}+\frac{1-w_{PHB}}{T_{g\;Plasticizer\;}}$$

where *w_PHB_* is the mass fraction of PHB, and *T_g_ _PHB_* and *T_g Plasticizer_* are the glass transition temperatures of PHB and the plasticizer used, respectively.

**Figure 6:**
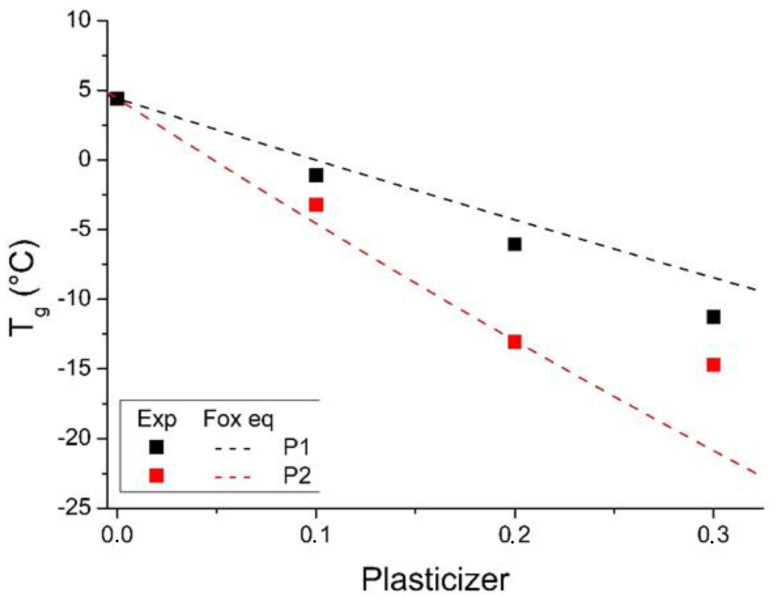
Experimental values of the change in T_g_ for PHB blends as a function of the concentration of the plasticizers. The dotted lines show the T_g_ theoretical model based on the Fox equation.

The resulting model obtained using the Fox equation along with the practical T_g_ results of the PHB blends is shown in Figure 6. The T_g_ values of P1 (−34.2 °C) and of P2 (−64.9 °C) were obtained using DSC.

As shown by the experimental data, adding both plasticizers causes a significant decrease in T_g_, which is more pronounced for PHB/P2. This indicates that the plasticizer interaction occurs in the amorphous phase of PHB. This effect has already been observed in the work of Bibers et al. [38].

The reduction in T_g_, and effective plasticization, occurs from the choice of plasticizer with a balance of molecular weight, spatial structure, and the content of functional groups [25]. In this case, the plasticizers used have aliphatic chains as spacers, providing structural mobility and the ester group as a linking segment with the polymer [39]. The highest C-O/C=O ratio in P2 demonstrated greater effectiveness in plasticizing PHB. It is possible to observe that the Fox model describes the variation in T_g_ well for both plasticizers up to a concentration of 20%. Comparatively, the best fit occurs in samples with P2, demonstrating a more significant interaction with the PHB matrix, also associated with a more pronounced plasticizing effect (greater reduction in T_g_) in the DSC results.

The T_g_ deviation between the data obtained experimentally and the theoretical model can be associated with the lack of miscibility in the polymer matrix [37]. Thus, these results indicate phase separation at high plasticizer concentrations (30%), as indicated by the more expressive mismatch of practical and theoretical T_g_ results for the plasticizer concentration of 30%.

### 3.3. Morphology

**Figure 7:**
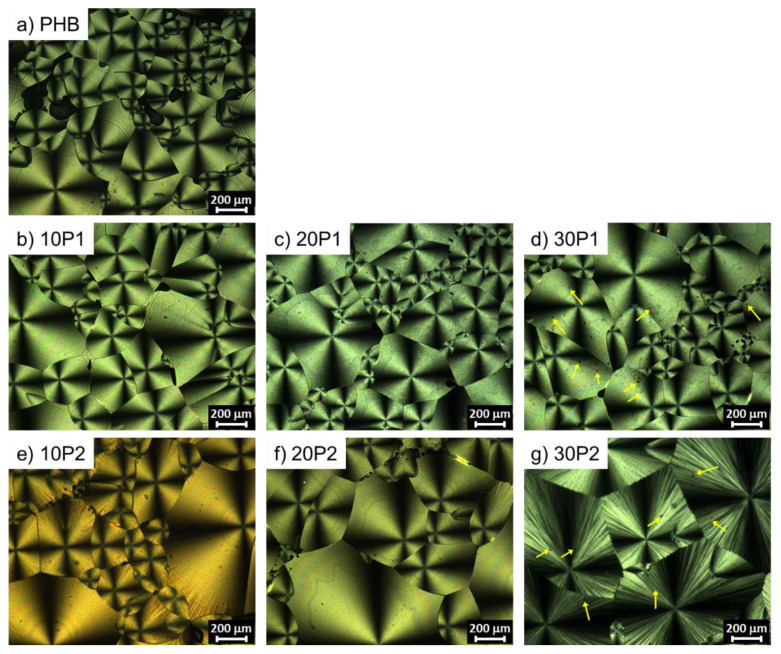
Polarized optical microscopy images of (**a**) PHB, (**b**) PHB/10P1, (**c**) PHB/20P1, (**d**) PHB/30P1, (**e**) PHB/10P2, (**f**) PHB/20P2 and (**g**) PHB/30P2 isothermally crystallized at 60 °C. Yellow arrows indicate dark spots.

Polarized optical microscopy (POM) was used to evaluate the effect of the addition of plasticizers on the spherulitic morphology of PHB. The samples were heated to 210 °C and cooled to 60 °C (isothermal crystallization temperature) to promote the samples’ crystallization. The POM images of the morphology obtained after crystallization are shown in Figure 7.

All samples showed large spherulites with the characteristic Maltese cross. Neat PHB under controlled conditions presents spherulites with sizes of 350–500 μm [40]. The presence of additives (nucleating agents) or plasticizers can change the size of the spherulites with variation in the nucleation rate [22,24,41].

POM image of PHB (Figure 7a) shows spherulites with size variation between 68 and 598 µm and a mean value of 209 µm. The PHB/P1 samples (Figure 7b–d) show a slight variation in the size of the spherulites, with mean values in the order of 249, 270, and 290 µm for concentrations of 10, 20, and 30 wt% of P1, respectively. The addition of P2 showed higher spherulites when compared with both PHB and PHB/P1. PHB/10P2 (Figure 7e) has spherulite size variation between 119 and 598 µm and a mean value of 274 µm. Samples with higher concentrations of P2 demonstrate an increase and a more uniform distribution of spherulites. PHB/20P2 (Figure 7f) and PHB/30P2 have mean sizes of 339 µm and 628 µm, respectively.

The addition of plasticizers increased the size of the spherulites. This behavior occurs proportionally to the plasticizer concentration, but its effect is more evident from adding P2, which has larger and more uniform spherulites. These results may be associated with the more significant influence that P2 has on the PHB structure compared to P1 and converge with the results presented by DSC, especially for the variation in T_c_. Thus, the addition of P2 caused a reduction in the nucleation rate and, consequently, promoted an increase in the diameter of the spherulites. A similar phenomenon is observed in work by Umemura et al. [22] with the addition of triethyl ci-treat in PHB. Furthermore, in the samples with 30 wt% of plasticizers, there was a higher incidence of dark spots through the spherulites (yellow arrows); this phenomenon is reported as amorphous phases due to phase separation [37].

### 3.4. Crystalline Structure

**Figure 8:**
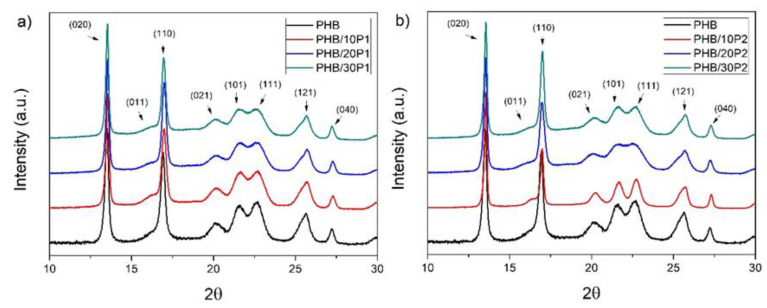
XRD spectra of PHB, PHB/P1 (**a**), and PHB/P2 (**b**) blends.

The analysis of the crystalline structure of neat PHB and its plasticized blends was performed by XRD, and the resulting diffractograms, with the assigned planes, are shown in Figure 8. The PHB and PHB blend diffractograms exhibited similar profiles corresponding to the orthorhombic unit cell [28,42] normally obtained for neat PHB.

As seen in Figure 8, the crystalline peaks present in the spectra are not modified by the addition of plasticizers, which corroborates the crystalline results obtained using DSC analysis. All samples presented two strong crystalline peaks at 2θ ≈ 13.5° assigned to the (020) plane and 2θ ≈ 17° to the (110) plane of the orthorhombic unit cell, while also containing a less intense peak set to the (021) plane (2θ ≈ 20°), indicating that the samples have a small amount of orthorhombic β-form crystals with zigzag conformation [28].

The X_XRD_ (%) values obtained from the XRD spectra have the same order of magnitude as those obtained from the DSC, but with different absolute values and behavior, as presented in Table 4. The X_XRD_ of PHB from XRD was 70.17%, whereas XDSC was 61.78%. While the addition of plasticizers to PHB slightly increased the X_DSC_ value, neither P1 or P2 content significantly altered this value. The addition of plasticizers caused a decrease in the X_XRD_, and the increase in plasticizer content resulted in the reduction in the crystallinity index calculated. The difference between the crystallinities reported by DSC and XRD can be associated with the difference between the methods; XRD emphasizes surface crystallinity while DSC represents bulk behavior [28].

### 3.5. Mechanical Behavior

**Figure 9:**
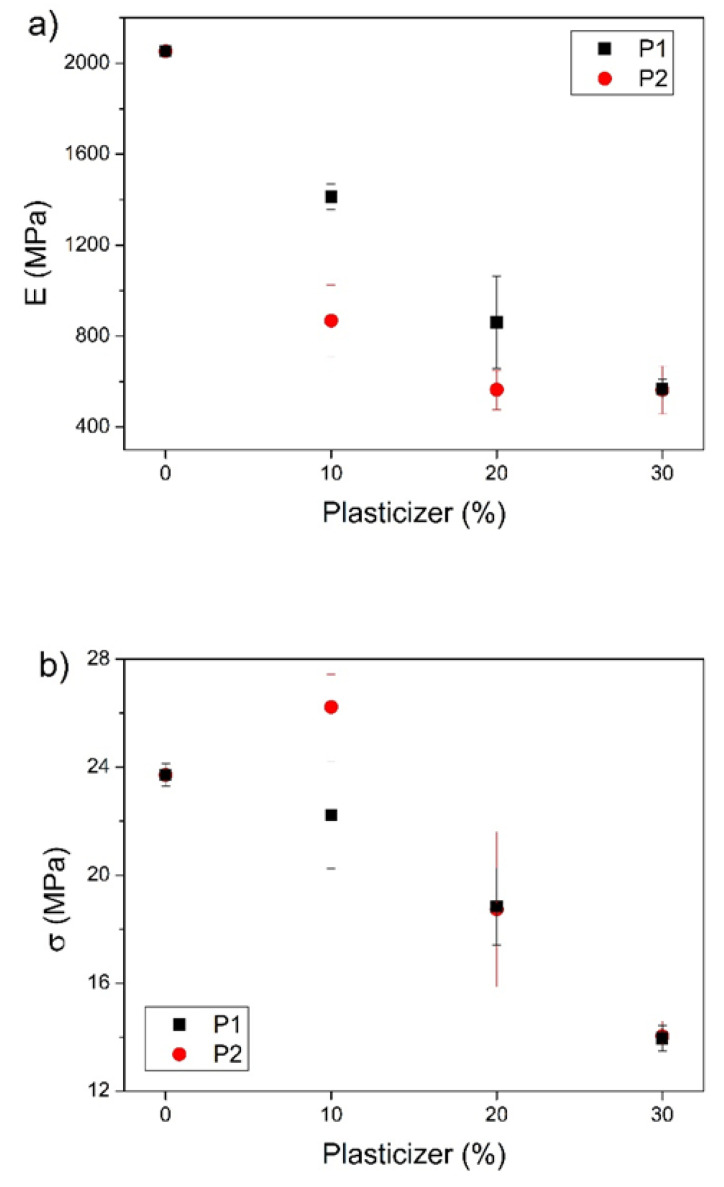

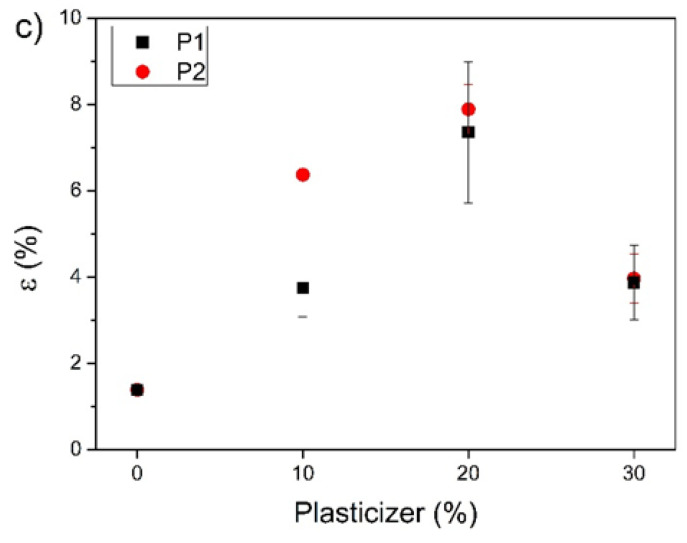
Tensile properties of PHB, PHB/P1, and PHB/P2 blends. (**a**) Elastic modulus (E); (**b**) tensile strength (σ); (**c**) elongation at break (ε).

The effect of the plasticizers’ concentration on the mechanical properties of PHB, i.e., elastic modulus (E), maximum tensile strength (σ), and elongation at break (ε), is shown in Figure 9. Neat PHB is a material with high rigidity due to its crystallinity [23]; it has a high elastic modulus and low elongation at break. Plasticizers with greater free volume than the polymer reduce the relative number of polymer–polymer contacts, providing the flexibility of the structure and thereby decreasing the rigidity of the three-dimensional structure, resulting in higher ε values [17,43].

As shown in Figure 9a, the addition of plasticizers caused a decrease in E. Initially, this behavior is more pronounced for P2; however, as plasticizer content increases, the E value for both starts to match, showing a decrease of 72% in E for blends with 30% of plasticizer when compared to neat PHB. However, the tensile strength (Figure 9b) only showed an increase for samples with 10% of P2 and no significant changes for PHB blends with 10% of P1. As the plasticizer concentration increases, the σ for both the PHB/P1 and PHB/P2 blends decreases.

The effects of plasticizer concentration on the elongation at break are seen in Figure 9c. The PHB ε value increases for all plasticizer concentrations studied in this work. For PHB blends with 10% plasticizer, for P2 there was an increase of 359% in the PHB elongation at break, while P1 showed an increase of 170%. At 20% of plasticizer, ε increases approximately 450% for both plasticizer–PHB formulations compared to neat PHB. At 30% plasticizer, there is a decrease in the maximum elongation compared to their value at 20%.

This behavior is also observed in other plasticized PHB systems [24,28,44] and is attributed to the high crystallinity of PHB, which hinders the diffusion of the plasticizer chains in the crystalline regions and causes a concentration saturation of plasticizer in the system, reducing its mechanical properties. These results support the phase separation hypothesis generated from the T_g_ results for samples with the addition of 30% plasticizer.

Thus, both oligomers P1 and P2 are effective when used as plasticizers for PHB, wherein P2 indicates better mechanical properties than P1. Depending on the desired properties of the final products, the optimal concentration of plasticizer could be selected with a critical concentration of 20%, as higher concentrations could present system saturation and the consequent deterioration of mechanical properties.

## 4. Conclusions

Polymers are indispensable materials, and their production, by volume, exceeds that of steel. To a large extent, they are used in short-lived, single-use applications such as packaging, and classic fossil plastics have two main drawbacks: their depletable feedstock and their longevity in the environment. “White littering” and microplastics have become a huge area of concern because plastics cause harm to the environment. Bioplastics, i.e., biobased and/or biodegradable materials, can be part of the solution towards a circular transition of plastics. PHA materials can play a pivotal role here because they are degradable in different environments, including challenging ones such as cold sea water. PHB is the simplest representative of PHA, and it resembles the commodity plastic PP (polypropylene) in most properties. However, PHB is stiff and brittle, with a small processing window which, coupled with higher material price, limits its application potential. What is needed is a more flexible PHB formulation. Work has been conducted on several copolymers and blends extensively. In this study, the authors have proposed a novel approach: they have developed and tested two linear-structured polyester oligomers as plasticizers for polyhydroxybutyrate (PHB) to positively alter its mechanical and thermal properties.

TGA demonstrated the excellent thermal stability of PHB–plasticizer mixtures, while DSC showed a reduction in T_g_ by 16 and 19 °C, and T_m_ by 5 and 4 °C for PHB/30P1 and PHB/30P2, respectively. The miscibility of the mixtures was qualitatively evaluated using T_g_ calculated with the Fox equation, which showed good miscibility for up to 20% (by weight) plasticizer. The POM images revealed increased spherulite size using P2, and emphasized its good interaction with PHB.

The best plasticizing effect occurred with the addition of P2, which had the highest C-O/C=O ratio: 2.226 versus 0.923 for P1. PHB/P2 presented an increase in tenacity and demonstrated an optimized concentration of 20%. With a concentration of 30% in both plasticizers, there was an indication of phase separation which resulted in the deterioration of mechanical properties.

Therefore, the aliphatic polyesters used provide the effective plasticization of PHB with superior thermal stability compared to low-molecular-weight plasticizers. It is assumed that this work contributes to the advancement of PHA formulation development by offering a route to improved material properties through novel, biobased and biodegradable plasticizing agents.

## Figures and Tables

**Table 1:** XPS parameters for plasticizers.

**P1**			
**C1s**	**eV**	**Area**	**O/C Ratio**
C-C	284.78	253,860.40	0.337
C-O	286.36	55,793.56	
C=O	288.71	49,916.54	
O1s			
C=O	531.92	201,597.11	
C-O	533.27	199,786.81	
**P2**			
**C1s**	**eV**	**Area**	**O/C Ratio**
C-C	284.72	153,483.66	0.306
C-O	286.26	120,512.52	
C=O	288.59	33,676.95	
HO-C=O	290.22	9883.34	
O1s			
C=O	531.80	85,402.08	
C-O	533.18	188,690.41	
HO-C=O	535.21	11,023.46	

**Table 2:** Thermal degradation parameters of PHB, PHB/P1, and PHB/P2 blends.

Material	T_5%_ (°C)	T_d1_ (°C)	T_d2_ (°C)
PHB	268.41	288.37	-
P1	243.48	377.63	-
P2	302.98	414.91	-
PHB/10P1	269.52	287.27	363.47
PHB/20P1	269.35	284.97	351.81
PHB/30P1	269.94	290.86	368.41
PHB/10P2	269.25	287.17	390.82
PHB/20P2	266.62	284.72	383.96
PHB/30P2	268.37	287.54	383.38

**Table 3:** Main parameters obtained from DSC analysis of PHB, PHB/P1, and PHB/P2. T_g_ is the glass transition temperature; T_c_ is the crystallization peak temperature; ΔH_c_ is the enthalpy of crystallization; T_cc_ is the cold crystallization peak temperature; ΔH_cc_ is the enthalpy of cold crystallization; T_m_ is the melting peak temperature; ΔH_m_ is the enthalpy of melting; X_DSC_ is the calculated crystallinity.

Material	T_g_ (°C)	T_c_ (°C)	ΔH_c_ (J g^−1^)	T_cc_ (°C)	ΔH_cc_ (J g^−1^)	T_m_ (°C)	ΔH_m_ (J g^−1^)	X_DSC_ (%)
PHB	4.38	68.11	55.53	55.71	2.75	175.24	90.20	61.78
PHB/10P1	−1.10	64.63	45.46	48.61	5.28	172.48	86.64	65.94
PHB/20P1	−6.06	66.04	37.90	46.12	6.05	171.21	76.80	65.75
PHB/30P1	−11.27	72.30	32.92	43.72	5.96	169.23	68.20	66.73
PHB/10P2	−3.23	53.64	31.24	51.09	12.68	174.40	82.38	62.69
PHB/20P2	−13.07	59.40	20.86	50.18	16.17	172.68	76.23	65.26
PHB/30P2	−14.71	59.47	22.09	49.23	11.22	171.21	64.56	63.17

**Table 4:** Calculated crystallinity index of PHB, PHB/P1, and PHB/P2 blends using DSC (X_DSC_) and XRD (X_XRD_) data.

Material	X_DSC_ (%)	X_XRD_ (%)
PHB	61.78	70.17
PHB/10P1	65.94	64.22
PHB/20P1	65.75	58.75
PHB/30P1	66.73	53.78
PHB/10P2	62.69	67.76
PHB/20P2	65.26	59.30
PHB/30P2	63.17	56.24

## Data Availability

The data presented in this study are available in this article.
